# Public Engagement in Health Priority Setting in Low- and Middle-Income Countries: Current Trends and Considerations for Policy

**DOI:** 10.1371/journal.pmed.1001495

**Published:** 2013-08-06

**Authors:** Katarzyna Bolsewicz Alderman, David Hipgrave, Eliana Jimenez-Soto

**Affiliations:** 1 School of Population Health, University of Queensland, Australia; 2 Nossal Institute for Global Health, University of Melbourne, Australia

## Abstract

David Hipgrave and colleagues argue that we must find more effective, equitable, feasible and affordable ways to engage the public in health priority setting in developing countries.

*Please see later in the article for the Editors' Summary*

**Summary Points**

- Many donors and low- and middle-income countries (LMICs) are now encouraging increased public participation in health sector priority setting (HPS).
- Using country examples, we demonstrate that despite many attempts, affordable, appropriate, and effective engagement of the public in that context remains elusive.
- Rather than mandating public participation in HPS, countries and donors should focus on building a policy environment that is conducive to grassroots initiatives and on strengthening the evidence for what works using small pilot studies.

## Public Engagement in Public Sector Activities

Democratization, rising literacy, the advent of the information age, and increasing connectedness are resulting in unprecedented opportunities for public participation in public affairs. In addition to promoting transparency and demonstrating inclusiveness, public consultation enhances ownership and resonates with the increasingly common decentralization of responsibility for social services to local authorities [1]–[5].

In the last decade, the World Bank alone spent almost $85 billion on local participatory development initiatives [6]. Examples of institutionalized public engagement in public sector activities in low- and middle-income countries (LMICs) include social audits to produce community-validated data for planning [7] and citizens' report and score cards to channel public feedback on the performance of public services [8],[9]. Such exercises can also be used to inform local budgeting and resource allocation [10],[11]. Even the campaign on post-2015 global development priorities [12] has proposed broad consultation involving the poor and the vulnerable, and recent commentaries on health sector regulation have called for broad public participation [13].

## Trends in Public Engagement in Health Priority Setting in Low- and Middle-Income Countries

Public engagement in priority setting is one example of broader initiatives to involve the LMIC public in development, and was included in the recent World Bank evaluation [6]. Health sector priority setting (HPS) usually involves major decisions on which services, programs, and population groups are prioritized for funding [14]. It is a complex and political exercise and can impact on the well-being of population groups affected by the choices made at each level.

High-income countries [4],[15]–[20], LMICs [1],[10],[20]–[25], and donors [6] have all recognized that the views and values of public beneficiaries of health services should be included in planning, including HPS. Public engagement in that context is thus encouraged or even prioritized in many nations. For example, authorities responsible for the introduction of new health technology have involved patient representatives and lay persons in the process [20]. Similarly, in many LMICs public engagement in HPS has been mandated at subnational level, or is increasingly promoted. In Uganda, nominated community members were recommended to represent the public on technical committees in health sector decision making [1]. In Kenya, local health workers develop an annual list of priority activities and targets, informed by the local community [21]. In Indonesia, *Musrenbang* is an annual, bottom-up participatory budgeting process during which residents prioritize community development options; it was created specifically to replace Indonesia's former centralized system [22]. In India, the National Rural Health Mission advocates for increased stakeholder and public engagement in priority setting at the village, sub-center, block, district, and state levels [23]. And a recent ordinance in the Philippines requires bottom-up planning for poverty alleviation to incorporate community and grassroots organizations' perspectives at the local government unit level [25].

## Is There Evidence to Guide Public Engagement in Health Priority Setting?

Notwithstanding these efforts and despite the expected benefits of public engagement in HPS, there is currently little evidence on how to undertake it effectively. Mitton and colleagues identified 15 different techniques through which the public in high-income countries was engaged in HPS (either at the national or local level) [15]. Some even involved deliberative methods, in which the public was engaged face-to-face over a period of time. However, only one third of over 170 studies assessed included some form of evaluation, and this mostly comprised process evaluation. (The World Bank review of its own Project Assessment Documents noted that only 40% of those with a focus on local participation included a monitoring system [6].) Only one study systematically compared the outcome of the approaches used, and concluded that deliberative engagement can affect the outcome, provided that the decision relates to a manageable issue affecting a specific population [26]. Ultimately, the study recommended a balanced approach of broad consultation with in-depth, deliberative engagement, but noted that evidence in support of any approach was weak.

In addition, the perceptions of the public on their roles in this process, and on methods for their engagement, have not been well assessed. Moreover, while the public may seek participation in HPS [1],[4] and indeed demand various roles according to the level and objectives of the process [18],[27], this may differ from what health practitioners and bureaucrats expect or want [27]. Not surprisingly then, it is difficult to form practical guidelines on whom to engage, when and in what role(s), and how to combine different stakeholders' inputs in HPS, especially given how context-specific these might be [5],[15],[18],[27],[28].

If the appropriateness and effectiveness of public engagement is unclear in high-income countries [4],[5],[15], the situation is even less certain in LMICs. Not only does the context differ greatly from that in high-income countries, but engaging the LMIC public in HPS in a meaningful, equitable way is also affected by various structural barriers including physical access, poverty, social exclusion, and the low social status of women in many settings. These barriers have the potential to result in “civil society failure.” As noted in the World Bank report [6], “organizing groups of people to solve market and government failures is itself subject to problems of coordination, asymmetric information and pervasive inequality” (p. 4).

For example, in a study in Uganda poverty was a practical determinant of whether people could participate at meetings; the patriarchal culture was intimidating for young people; and community members felt the local councils were not interested in their perspective, and that only those with something to offer (the rich) were actively mobilized for planning meetings [1]. In Kenya [21] and Tanzania [24], although national policy mandates community engagement in local health planning, community representatives were disenfranchised by a lack of information, facilitation and time constraints, and an overarching disconnect between local and national priorities. In decentralized Pakistan, the frequent transfer of staff, capture of HPS by local elites, and corruption were among many factors that contributed to the failure of mandated, bottom-up resource allocation and budgeting [29]. In settings where women are disempowered and the poor marginalized, mandating public participation in any public process does not change the effective exclusion of these groups, and does not reliably improve the outcomes of the decision-making process [6].

It seems that in many LMICs, even if the public has a constitutional right to participate and expresses interest in being involved, the processes for public participation do not function as intended. Moreover, although public engagement can be costly and time consuming, little public input is actually incorporated into plans and budgets. For political, practical, or cultural reasons and in the absence of effective oversight, most HPS in LMICs remains dominated by the “executive” and appointed authorities [1],[21],[24]. To summarize, although public participation in that context is perceived as the right thing to do, based on current evidence it seems unfeasible and unlikely to result in helpful outcomes.

## Where to from Here?

Weak evidence should not delay efforts to increase public involvement in HPS [4]. However, ongoing token efforts at public engagement may damage trust, particularly if the process ultimately favors the priorities of wealthier households, groups with a higher status, or those with vested interests. Given that bottom-up planning and budgeting in LMICs is widely encouraged, and the high financial and social risks associated with this, it is imperative to document and evaluate related processes and outcomes [5],[6],[15],[16],[18]. So far, the literature has focused on the intrinsic values of public engagement in HPS (e.g., transparency, inclusiveness, social capital) instead of pragmatic but important considerations relating to what works, how to engage the most disadvantaged groups, whether the public wants to be engaged and by what means, how to monitor and evaluate related processes, and what has been achieved and at what cost [5],[15]. Going forward, pilots or assessments of current activities with a strong evaluation component are needed, first to capture the means of public engagement at the local level, and second to identify which approaches better reflect the public voice and yield more efficient, equitable, and effective resource allocation. Documentation should consider whether and how structural, political, and cultural barriers to public engagement were addressed, including the issues of marginalization of certain groups, corruption, elite capture, and public distrust. Such activities would ideally not be undertaken as a time-limited “project,” but in the context of national efforts to improve collaborative governance and stewardship of public processes, including in the health sector [30].

Whether guidelines or mandates on public engagement in HPS should be developed remains questionable. The recent World Bank report indicates that induced, especially donor-driven, local participatory development initiatives have not in general been beneficial [6]. Accordingly, it seems more appropriate for LMICs to create a policy environment that provides incentives for community participation in HPS, and enables it to evolve organically, based on feedback, refinement, and ongoing evaluation against agreed indicators. This approach is supported by experts in the field of priority setting, who now recommend generic principles and performance indicators based on locally defined objectives and standards [16]. Certain mechanisms currently used to give voice to the public in LMICs (e.g., social audits, citizens' report cards and score cards) could lend themselves to HPS and also to the evaluation of such initiatives. In addition, applying selected practical measures seen across these mechanisms [31],[32], such as publicizing decisions on resource allocation and making available an independent appeal process, would help create an environment that is conducive to public engagement in that context. Evaluation and research should also further assess the capacity of lay people to make informed decisions on HPS [30], how to integrate their inputs and those of others, and the conditions required for these inputs to inform actual resource allocations.

To summarize, the new global recommendations on collaborative governance in the health sector [33] may inadvertently pressure LMICs to engage the public in health planning, including HPS. However, the weak evidence on how to do this, the substantial resources required, and risks associated with such processes suggest that countries mandating public engagement in that context should reconsider their related policies. Moreover, the resources and efforts from development partners currently invested in advocating for public engagement in HPS may be better spent on strengthening the evidence for what works within the realities of LMICs, using small-scale, community-driven trials. National authorities should aim to create an environment that is conducive to such public engagement by using practical measures such as publicizing the decisions on resource allocation and having a mechanism for appeal.

## Abbreviations

HPS: health sector priority setting
LMICs: low- and middle-income countries
